# Advances in the production of fungi-derived lignocellulolytic enzymes using agricultural wastes

**DOI:** 10.1080/21501203.2023.2253827

**Published:** 2023-09-13

**Authors:** Jiaqi Huang, Jianfei Wang, Shijie Liu

**Affiliations:** Department of Chemical Engineering, SUNY College of Environmental Science and Forestry, Syracuse, NY, USA

**Keywords:** Lignocellulolytic enzyme, fungi-derived, agricultural wastes, fermentation, genetic modification

## Abstract

Lignocellulolytic enzymes play an important role in various industrial applications as well as the sustainable valorisation of lignocellulosic materials. Enzyme production using lignocellulosic fungi has shown great advantages such as high enzyme diversity, high production efficiency, and the availability of solid waste as raw materials. Agricultural waste, an abundant and non-food competitive feedstock, can be used to produce fungal lignocellulolytic enzymes. Pretreatment helps break down the complex structure of the raw material, thereby significantly improving product yield but also requiring more energy consumption. Multiple fermentation technologies, including submerged fermentation, solid-state fermentation, and co-culture, can be used for producing lignocellulolytic enzymes. Process optimisation may promote the yield and productivity of such enzymes without additional investment. Genetic engineering is also useful for enhancing enzyme production to meet industrial requirements. This review summarises the research progress in the fungal production of lignocellulolytic enzymes from various agricultural wastes via advanced fermentation strategies. It aims to provide technical references for the scale-up production of fungal lignocellulolytic enzymes.

## 1. Introduction

The global enzyme market size has been growing and is expected to reach $8.7 billion by 2026 (Dewan 2021) owing to increasing demands for adapting environmental-friendly solutions and finding sustainable alternatives to fossil fuels. Lignocellulosic enzymes account for more than 20% of enzyme sales in the global market (Leite et al. 2021). Combined with specific techniques, these enzymes have shown significant potential in many industrial and environmental applications, such as paper making, food processing, and pollutant degradation (Chukwuma et al. 2020; Saldarriaga-Hernández et al. 2020; Saini and Sharma 2021). Furthermore, lignocellulolytic enzymes can depolymerise lignocellulose into fermentable sugars under mild conditions. As a type of microbial enzyme, they are also highly resistant to the inhibitory effects of other microbial metabolites (Hosseini Koupaie et al. 2019). These properties have aroused enormous research interest in utilising lignocellulolytic enzymes to facilitate the bioconversion of lignocellulosic materials into value-added products, such as biofuels (Raud et al. 2019) and biopolymers (Wang et al. 2021; Wang et al. 2023). On the other hand, lignocellulose is a natural and ideal inducer for the production of lignocellulolytic enzymes. Agricultural residues are the representative form of lignocellulosic biomass waste. Large amounts of agricultural waste have been generated due to the gradually expanded agricultural production with population growth, resulting in negative environmental impacts. Utilising them as feedstocks to produce lignocellulosic enzymes is a promising approach to valorising these wastes while easing the environmental pressure of waste disposal.

Lignocellulolytic enzymes can be produced extracellularly by both bacteria and fungi. Compared with lignocellulolytic bacteria, the lignocellulolytic fungus can produce lignocellulolytic enzymes with higher diversity due to their more powerful metabolic systems for extracellular enzyme synthesis (Andlar et al. 2018). They also have higher adaptability to low-moisture environments to produce lignocellulolytic enzymes from solid wastes with minor pretreatment. It is conducive to improving enzyme production efficiency and reduces the risk of contamination and enzyme degradation (Leite et al. 2021). Lignocellulolytic fungi are grouped based on their different effects and preferential degradation to lignocellulosic polymers. White-rot fungi show excellent efficiency in the degradation of lignin. Soft-rot fungi have the highest cellulolytic and hemicellulolytic enzyme system, followed by white-rot fungi and brown-rot fungi (Sista Kameshwar and Qin 2018). Table 1 lists the enzyme-producing fungi, enzyme characteristics, and recent applications of various lignocellulolytic enzymes. However, their widespread industrial applications have been facing the major challenge of insufficient enzyme productivity and selectivity. Therefore, comprehensive knowledge is still needed to improve production efficiency by optimising fermentation conditions, simplifying processes, and exploring new technologies.

**Table 1.** Recent applications of various lignocellulolytic enzymes from different wild-type fungal cultures.

Enzyme	Fungal culture	Molecular weight	Optimal temperature and pH for enzyme reaction	Applications	References
Lignin peroxidases	*Phanerocheate chrysosporium*	46 kDa	-	Biodegradation of toxic synthetic polymer waste	Khatoon et al. 2019
	*Paecilomyces* sp.	38 kDa, 40 kDa	20–30 °C, pH 2–3	Effluent treatment in pulp and paper industry	Singh et al. 2016
Manganese-dependent peroxidases	*Trametes pubescens*	55 kDa	40 °C, pH 5	Biodegradation of lignin, decolourisation of textile-dyes	Rekik et al. 2019
	*Cerrena unicolour*	45 kDa	60 °C, pH 4.5	Decolorization of synthetic dyes, bleaching of denim in textile industry	Zhang et al. 2018
Laccase	*Thielavia* sp.	70 kDa	70 °C, pH 5	Decolorization of anthraquinone-type dye without redox mediator	Mtibaà et al. 2018
	*Albifimbria viridis*	66 kDa	25 °C, pH 5	Removal of heavy metal from pollutants, biodegradation of tannin	Ahmadi Khozani et al. 2021
	*Penicillium chrysogenum*	68 kDa	≥80 °C, pH 5.5	Hydrolytic de-gradation of chemical pollutants	Senthivelan et al. 2019
Endoglucanase	*Thermoascus aurantiacus*	35 kDa	70 °C, pH 4	Improvement of productivity and nutritive quality in food industry, enhancement of cellulosic garments in textile industry	Dave et al. 2015
	*Fomitopsis meliae*	-	70 °C, pH 4.8	Refinery and saccharification of biomass in food, animal feed, paper, and pulp industries	Patel et al. 2021
	*Stachybotrys microspora*	55 kDa	50 °C, pH 7	Detergents, textiles and saccharification of lignocelluloses in bio-refinery processes and crop waste management	Ben Hmad et al. 2017
Exoglucanase	*Meyerozyma* sp.	70 kDa	70 °C, pH 5	Enzymatic hydrolysis of cellulose feedstock for renewable bioethanol production	Kuo et al. 2015
	*Aspergillus fumigatus*	-	55 °C, pH 4.8	Enzymatic hydrolysis of feedstock to produce organic acids and other chemicals	Mahmood et al. 2013
Glucosidase	*Aspergillus unguis*	10 kDa	60 °C, pH 6	Enzymatic saccharification of biomass	Rajasree et al. 2013
	*Lichtheimia ramosa*	-	65 °C, pH 5.5	Production of bioethanol, improvement of food and beverage quality	Garcia et al. 2015
	*Penicillium piceum*	80 kDa	60 °C, pH 6	Enzymatic hydrolysis of lignocellulosic biomass	Gao et al. 2014
Xylanase	*Coprinellus disseminatus*	43 kDa	55 °C, pH 6.4	Production in thermo-alkali-tolerant environment, pulp, and paper industry	Agnihotri et al. 2010
	*Aspergillus foetidus*	29.9 kDa	50 °C, pH 5.3	Production of value-added food ingredient from agro-residues	Chapla et al. 2013
	*Myceliophthora heterothallica*	27 kDa	65–70 °C, pH 4.5	Pproduction of xylooligosaccharides for use as prebiotics	de Oliveira Simões et al. 2019
Mannanase	*Aspergillus oryzae*	34 kDa	60 °C, pH 5	Bio-bleaching of pulp, augmentation of detergent, upgradation of feed quality, oil drilling	Jana et al. 2018
	*Penicillium chrysogenum*	30 kDa	50 °C, pH 6	Biobleaching of pulp, production of mannooligosaccharides in food industry	Shalaby et al. 2017
Arabinanase	*Aspergillus tubingensis*	-	-	Fruit juice processing, wine production	Okado et al. 2020
	*Aspergillus thermomutatus*	-	-	Deconstruction of lignocellulosic material	Benassi et al. 2014
Galactosidase	*Aspergillus niger*	76 kDa	50 °C, pH 5	As additive in food processing	Martarello et al. 2019
	*Aspergillus terreus*	42 kDa	40 °C, pH 6	Development of prebiotics to lactose intolerance patients	Vidya et al. 2020
Glucuronidase	*Penicillium purpurogenum*	70 kDa	40 °C, pH 6	Highly selective hydrolysis of glycyrrhizin to glycyrrhetinic acid monoglucuronide	Zou et al. 2013
	*Chaetomium globosum*	-	-	Production of glycyrrhetic acid 3-O-mono-β-D-glucuronide	Gao et al. 2021

- Not mentioned in the study.

This review discussed the recent studies on producing fungi-derived lignocellulolytic enzymes from various agricultural wastes. The pretreatment of the raw materials was also included. In addition, the significant enhancements in enzyme production by applying advanced fermentation technologies and strain modifications were addressed.

## 2. Lignocellulolytic enzymes

**Figure 1.**
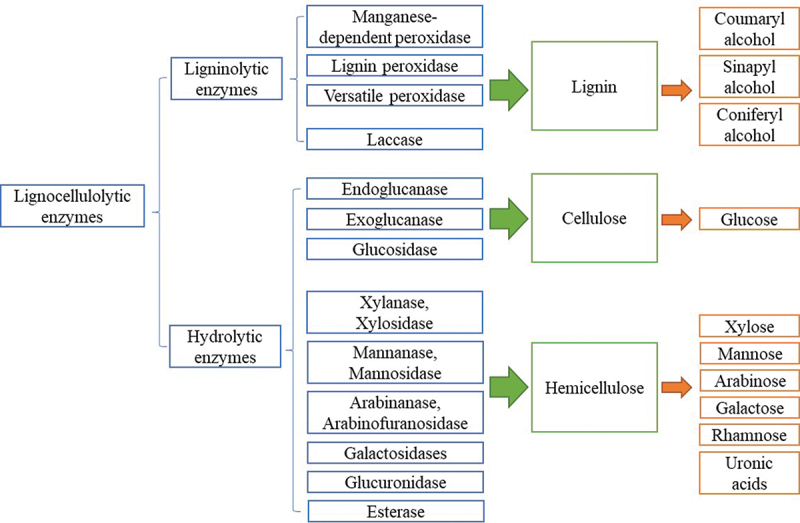
Overview of lignocellulolytic enzymes in lignocellulose degradation.

Lignocellulose mainly contains 5%–30% lignin, 20%–35% hemicellulose, and 35%–50% cellulose. The proportion of major components and the composition of their monomers may vary significantly in different plants. Therefore, the complete depolymerisation of lignocellulosic materials relies on the synergistic action of various lignocellulolytic enzymes. Based on the target substrate, these enzymes can be divided into ligninolytic enzymes and hydrolytic enzymes. The ligninolytic enzymes work on the disruption of lignin, whereas the hydrolytic enzymes are responsible for the hydrolysis of carbohydrate polymers. Figure 1 shows an overview of lignocellulolytic enzymes involved in the degradation of lignocellulose.

### 2.1. Ligninolytic enzymes

Lignin is an amorphous phenolic polymer with a complex crosslinked structure composed of phenylpropane derivatives. Lignin in different plants contains different percentages of monomers, linked to each other mainly by carbon-carbon bonds or ester bonds (Ralph et al. 2019). Lignin degradation is an oxidative process involving two groups of enzymes: Peroxidase and laccase. Peroxidase requires hydrogen peroxide as the electron acceptor to initiate the catalysing of non-phenolic compounds. Laccase uses oxygen as the oxidant to oxidise both phenolic and non-phenolic compounds (Chio et al. 2019).

### 2.2. Hydrolytic enzymes

Cellulose and hemicellulose are the two major carbohydrate polymers in lignocellulose. Cellulose is a linear polysaccharide consisting of hundreds or thousands of glucose molecules linked by β-1,4-glycosidic bonds (McNamara et al. 2015). In a typical cellulase system, endoglucanases act on the amorphous regions of cellulose and cleave glycosidic bonds internally, while exoglucanase act on the crystalline regions of cellulose to release β-cellobiose. The generated oligomers and cellobiose are then hydrolysed to glucose by glucosidase (Barbosa et al. 2020). Hemicellulose is a heterogeneous polymer composed of different five- and six-carbon sugars linked by β-1,3- and β-1,4-glycosidic bonds, and its branch chains may be characterised by uronic acids. In addition, hemicellulose may be closely bound to lignin and cellulose by aromatic ester and hydrogen bonds, respectively (Ge et al. 2018; Rao et al. 2023). Xylanases hydrolyse xylan and produce xylo-oligomers, which are hydrolysed by xylosidases to release xylose. Similarly, mannanases and mannosidases act on mannan together to release mannose. Other accessorial enzymes also play an important role. Arabinofuranosidase hydrolyzes the covalent bonds between arabinose and xylose. Esterases cleave the ester bonds uronic acids and oligomers (Houfani et al. 2020).

## 3. Fungal production of lignocellulolytic enzymes from agricultural wastes

### 3.1. Food crop waste

When food crops are harvested and processed, unwanted parts, such as stems, leaves, and husks, are discarded as wastes. Currently, wheat bran is one of the main substrates for the fungal production of various lignocellulolytic enzymes. By cultivating *Myceliophthora thermophila* (*M*. *thermophila*) with wheat bran, da Rosa-Garzon et al. (2022) reported the production of cellulases (309.8 U/g, Day 1), xylanase (4,105 U/g, Day 1) and glucosidase (178.4 U/g, Day 2). Garcia et al. (2015) obtained high production of glucosidase (274 U/g) from wheat bran by *Lichtheimia ramose*. The produced enzyme remained stable at a wide range of pH (3.5–10.5) and highly active under up to 10% ethanol. *Cotylidia pannosa* can be cultivated on wheat bran to produce an efficient lignocellulolytic enzyme cocktail containing cellulase (20 U/mL), xylanase (17 U/mL), and laccase (13 U/mL) (Sharma et al. 2016). Besides, rice waste biomass was identified as an ideal substrate for Streptomyces sp. to produce endoglucanase (132.6 U/gds), exoglucanase (14.6 U/gds), cellobiase (125.6 U/gds), and xylanase (342.5 U/gds). The enzyme complex had good resistance to high pH (5–8), high temperature (50–80 °C), and organic solvents (Saratale et al. 2017). Rice husk also showed good potential in laccase production (6.9 U/mL) by *Trametes versicolor* (*T*. *versicolor*) (Perdani et al. 2020). *Auricularia auricula* (*A*. *auricula*) can utilise alkali-treated corn stalk to generate glucosidase (2.92 IU/g, Day 6) and exoglucanase (2.92 IU/g, Day 8) (Lu et al. 2022). Astolfi et al. (2019) compared the cellulolytic enzyme production by *Trichoderma reesei* (*T*. *reesei*) among four different wastes, and soybean hulls were found to provide maximum activity xylanase (1,130.70 U/g, Day 7), in addition to endoglucanase (6.72 U/g, Day 15) and exoglucanase (5.45 U/g, Day 3). Ozcirak Ergun and Ozturk Urek (2017) investigated the ligninolytic enzyme production from potato peel waste by *Pleurotus ostreatus* (*P*. *ostreatus*). The highest laccase activity (6,708.3 U/L, Day 17) was achieved with dry peels, while the highest MnP activity (2,503.6 U/L, Day 17) was determined in neutralised fresh peels. Martarello et al. (2019) obtained galactosidase with the activity of 24.64 U/mL after culturing *Aspergillus niger* (*A*. *niger*) on soybean residues for 7 days.

### 3.2. Cash crop waste

Cash crops, also known as “technical crops”, generally refer to crops with relatively high value as industrial raw materials. In the palm oil industry, a vast amount of oil palm residues is generated in oil palm plantations and oil palm refineries. Taking oil palm fruit bunches as the substrates, *Aspergillus* sp. produced xylanase (54.32 U/g, Day 8), lignin peroxidase (13.41 U/g, Day 8) but low activity cellulase (Orozco Colonia et al. 2019). Ezeilo et al. (2020) cultivated *Trichoderma asperellum* (*T*. *asperellum*) and *Rhizopus oryzae* (*R*. *oryzae*) on oil palm leaves. *T*. *asperellum* generated endoglucanase (59.64 U/g, Day 4), exoglucanase (9.58 U/g, Day 4), glucosidase (118.1 U/g, Day 4) and xylanase (175.91 U/g, Day 5). *R*. *oryzae* generated the same enzyme cocktail but all at lower levels and required a longer cultivation period to reach maximum activity, endoglucanase (41.62 U/g, Day 6), exoglucanase (9.58 U/g, Day 5), glucosidase (113.07 U/g, Day 5) and xylanase (162.68 U/g, Day 6). Oil palm decanter cake after the refining process can also be used by *Pseudolagarobasidium* sp. to yield laccase (5.841 U/gds, Day 7) and manganese peroxidase (5.156 U/gds, Day 7) (Thamvithayakorn et al. 2019). Sugarcane bagasse, the main by-product of sugarcane sugar production, is a sustainable biomass resource rich in both sugar and fibre. Namnuch et al. (2021) confirmed that sugarcane bagasse was a good source for producing endoglucanase (1.27 U/mL), cellulase (0.72 U/mL), and xylanase (376.81 U/mL), after 14 days of fermentation by *Aspergillus flavus* (*A*. *flavus*). Another strain, *A*. *niger*, was evaluated on sugarcane bagasse and brewery spent grain, showing the highest activity for cellulase (6.23 U/gds, Day 5) and xylanase (1,400.80 U/gds, Day 5) on brewery spent grain and sugarcane bagasse, respectively (Moran-Aguilar et al. 2021). Ravindran et al. (2019) used spent coffee waste as the sole carbon source for xylanase production by *A*. *niger*, with the activity of 4,649 IU/gds on Day 5. Favaro et al. (2020) identified the best ratio of coffee waste and wheat bran as 1:1 for the highest production of mannanase (45 IU/g, Day 5) by *A*. *niger*. By combining olive pomace with winery waste, Filipe et al. (2020) made it possible to achieve the maximum production of xylanase (189.1 U/g, Day 7), cellulase (56.3 U/g, Day 4) and glucosidase production (10.9 U/g, Day 2) by *Aspergillus ibericus*.

### 3.3. Fruit waste

Fruit wastes, including stems, leaves, peel or shell, seeds, and bagasse, usually contain a higher number of fermentable sugars than crop wastes. Backes et al. (2022) studied the laccase production from pineapple crowns by *T*. *versicolor* and obtained a maximal laccase activity of 60.73 U/g in 7-day cultures. After 21-day cultivation of *Trametes hirsute* on pineapple leaf waste, extracellular laccase enzyme production reached a maximum activity of 3,003.2 U/mL (Chablé-Villacis et al. 2021). Gooruee et al. (2022) examined different species of *Trichoderma* fungi for a 2-day enzyme production from lemon peels. *Trichoderma afroharzianum* showed the highest activity of exoglucanase (5.42 U/mL), endoglucanase (6.02 U/mL), total cellulase (10.96 U/mL), and xylanase (4.11 U/mL), while *Trichoderma lixii* showed the highest activity of glucosidase (10.17 U/mL). Akpinar and Ozturk Urek (2022) directly applied peach waste for the lignocellulolytic enzyme production by *Pleurotus eryngii* (*P*. *eryngii*), namely manganese peroxidase (476.04 U/L), lignin peroxidase (895.80 U/L), endoglucanase (11.55 U/mL), xylanase (3.27 U/mL), exoglucanase (4.42 U/L), glucosidase (34.77 U/L). A similar enzyme cocktail was successfully produced by the same *P*. *eryngii* strain using cherry waste (Akpinar and Ozturk Urek 2020). *A*. *niger* was able to grow on melon residues and synthesise endoglucanase (1.21 U/mL, Day 2), xylanase (11.00 U/mL, Day 8), and laccase (18.23 U/mL, Day 5) (Rodríguez-Luna et al. 2022). When subject to a 3-day fermentation on passion fruit peel, *A*. *niger* produced exoglucanase (1.243 U/mL) and endoglucanase (1.714 U/mL) (Silva et al. 2019). de Oliveira Júnior et al. (2022) reused guarana processing residues to produce endoglucanase (0.84 U/g, Day 8), xylanase (1.00 U/g, Day 7), laccase (176.23 U/mL, Day 5) in addition to phenolic compounds. de Almeida Antunes Ferraz et al. (2018) fermented yellow mombin residue using *Penicillium roqueforti*, resulting in a crude enzyme extract rich in xylanase (14 IU/g).

### 3.4. Forestry waste

Forestry wastes are generated from forest harvesting and regenerating, for example, wood chips, bark, sawdust, timber slash, and mill scrap. They are lignocellulosic wastes with a relatively high content of lignin. Sunardi et al. (2016) studied the lignocellulolytic enzyme activity during the degradation of *Picea jezoensis* (spruce) wood by *Porodaedalea pini*. Ligninolytic enzymes generally had low activity, laccase (0.044 nkat/mg, Day 30), lignin peroxidase (0.368 nkat/mg, Day 120), and manganese peroxidase (2.100 nkat/mg, Day 120). Xylanase (89.788 nkat/mg), endoglucanase (70.425 nkat/mg), and exoglucanase (15.559 nkat/mg) activities reached their maximum levels on Day 60. Laccase activity from *P*. *ostreatus* and *Flammulina velutipes* (*F*. *velutipes*) was investigated with *Populus beijingensis* (hybrid poplar). Maximum laccase activity for both strains was observed on Day 7, except that *P*. *ostreatus* had a much higher activity (303.52 U/L) than *F*. *velutipes* (40.62 U/L) (An et al. 2021). Among the single cultivations of *Gloeophyllum sepiarium* (*G*. *sepiarium*) on birch, spruce, and pine wood, pine wood as the sole carbon source gave the most active endoglucanase (2.2 µkat/L, Day 24) and xylanase (6.2 µkat/L, Day 24). The highest activities of glucosidase (~0.06 µkat/L, Day 24), mannosidase (~0.018 µkat/L, Day 24), and xylosidase (~0.015 µkat/L, Day 24) were detected on birch, while the highest activity of galactosidase (0.18 µkat/L, Day 35) was on spruce. The ligninolytic enzyme activities were almost flat in all hardwood and softwood cultivations (Sugano et al. 2021). The research of Vázquez-Montoya et al. (2020) demonstrated that *Penicillium funiculosum* displayed the highest activities of endoglucanase (1,683 U/L, Day 4), exoglucanase (1,700 U/L, Day 4) and glucosidase (924 U/L, Day 6) on *Moringa oleifera* (benzolive tree) straw. Qi et al. (2022) compared the biodegradation abilities of serval fungi species in *Dendrocalamus sinicus* (bamboo) sawdust. The white-rot fungus *T*. *versicolor* produced the full spectrum of lignocellulolytic enzymes, exoglucanase (40.12 U/mL), endoglucanase (~10 U/mL), hemicellulose (~25 U/mL), laccase (~440 U/mL), manganese peroxidase (500 U/mL), and lignin peroxidase (~330 U/mL). The brown-rot fungi *Gloeophyllum trabeum* and *Rhodonia placenta* had higher hydrolytic enzyme activities, exoglucanase (55.18 and 180.26 U/mL), endoglucanase (~130 and 180.26 U/mL), hemicellulose (186.6 and 169.65 U/mL), but much lower ligninolytic enzyme activities, laccase (<3 U/mL), (<5 U/mL), lignin peroxidase (~58 and ~33 U/mL).

### 3.5. Pretreatment of agricultural waste

One of the major challenges in producing lignocellulolytic enzymes from agricultural wastes is the highly complex and recalcitrant structure of lignocellulose. Typically, biomass is pretreated to disintegrate the lignin from cellulose and hemicellulose, allowing the microorganism to access the sugars present within the holocellulose fraction. In addition, agricultural residue is a mixed waste of non-uniform sizes and compositions, which may contain many factors that inhibit the growth of fungi. Altering these inhibitors by pretreatment has significant impacts on fungal cultivation. The common pretreatment approaches are classified into physical, chemical, and physicochemical. Physical pretreatment techniques, including mechanical, ultrasonic, microwave, and thermal pretreatment, are used to reduce the particle sizes and promote the efficiency of subsequent treatments. Chemical pretreatment applies acids, alkalis, oxidation, or organic solvent to disrupt the lignocellulose structure and enhance cellulose digestibility. Acid is commonly used to dissolve hemicellulose and convert the dissolved hemicellulose into fermentable sugars. Concentrate acid forms inhibitors and causes reactor corrosion. Thus, dilute acid is preferred, with a rapid reaction rate favouring continuous biomass processing. Alkaline is another available reagent for biomass pretreatment. It can remove lignin and promote deacetylation and uronic acid removal from hemicellulose. In addition, the mixed reagents can improve the performance of pretreatment. For example, green liquid, a mixture of sodium carbonate and sodium sulphide, increased the conversion rate by reducing cellulose crystallinity and polymerisation degree (Malik et al. 2022). To further improve the biodegradability of agricultural wastes, physicochemical pretreatment methods combine the advantages of physical and chemical treatments, such as steam explosion, alkali-heat pretreatment, and ammonia fibre expansion (Awogbemi and Von 2022). Lu et al. (2022) compared the cultivations of *A*. *auricula* on corn stalks pretreated with alkali, alkali-ozone, ozone, and high-temperature water (control). The alkali pretreatment group contributed to the maximum growth of mycelium (1.2 times of control) and the highest activity of glucosidase (3.4 times), whereas the ozone treatment group showed the highest activity of laccase (2.1 times). Perdani et al. (2020) proposed that the laccase yields by *T*. *versicolor* from cornstalk, rice husk, and bagasse after steam explosion were 1.61, 1.86, and 1.66 times as high as those without pretreatment. The production of cellulase by *A*. *niger* using brewery spent grain autoclaved at 130 °C for two hours was 1.8 times that of untreated (Moran-Aguilar et al. 2021). However, the additional energy consumption and waste pollutants caused by the pretreatment process cannot be ignored. Blindly pretreating raw materials for optimal enzyme production at all costs is impractical and unsustainable.

## 4. Fermentation technologies for enzyme production

The purpose of developing a fermentation process is to increase productivity by adjusting fermentation conditions without adding additional processes, thereby reducing production costs, and maximising resource utilisation. Application of appropriate fermentation strategies can facilitate the fungal transformation of agricultural wastes, leading to higher yields of target lignocellulolytic enzymes.

### 4.1. Submerge fermentation (SmF) and Solid-State fermentation (SSF)

SmF and SSF are the most used fermentation technologies for lignocellulolytic enzyme production by microbes. SmF is a process where microorganism grows in the liquid broth, while SSF takes place in the absence or near absence of free water. Since SmF is technically easier to implement with process controls, it has been more readily available for industrial production. The better control of fermentation parameters and nutrient composition in SmF contribute to a high stability of enzyme production. On the other hand, SSF offers several advantages for those fungi species that struggle in liquid environments. The risk of contamination is substantially reduced in SSF due to the low moisture content. For fermentations with solid agricultural waste, using SSF may simplify the pretreatment of feedstocks, resulting in reduced energy consumption and cost savings. However, for any enzyme production of interest, there is no magic recipe that works for all strains. Liu et al. (2020) investigated the expression of lignocellulolytic enzymes from corn stover powder by *Phanerochaete chrysosporium* (*P. chrysosporium*). Most enzyme activities from SSF were 2 to 2.3 times as high as those from SmF, except xylanase activity from SSF was 20% lower. Both enzyme cocktails showed similar hydrolysis results at high substrate loading, but the weaker enzyme cocktail from SmF was more effective at low substrate loading due to the higher ratio of carbohydrate-binding enzymes in SmF. Elegbede and Lateef (2018) selected eight fungi strains to produce xylanases from corncob in SmF and SSF. SSF yielded higher xylanase activity (1.1 to 3.4 times that of SmF) in half of the strains, *Aspergillus fumigatus*, *Botryodiplodia* sp., *A*. *flavus*, *A*. *niger*. In the other half, up to 80% of xylanase activity was lost in SSF. Enzyme productions by *P*. *ostreatus* were compared in SSF and SmF using grape pomace as the substrate. The highest endoglucanase activity of 0.93 U/g was obtained in SmF (0.07 U/g in SSF), whereas the maximum laccase activity of 26,247 U/g was observed in SSF (4,447 U/g in SmF) (Papadaki et al. 2019). In sequential SSF-SmF of palm empty fruit bunches by *Aspergillus tubingensis* (*A*. *tubingensis*), activities of cellulase and xylanase (89.6 U/g and 196.8 U/g) were significantly higher than in SSF alone (40.09 U/g and 62.43 U/g) or SmF alone (66.02 U/g and 158.5 U/g) (Intasit et al. 2021).

### 4.2. Co-cultivation

In nature, the degradation of lignocellulosic materials is a long process accomplished by a variety of organisms with different specialities in degrading different portions of lignocellulose. Co-cultivation is a technique of growing two or more fungi with complementary enzyme activities to create a more diverse and robust enzyme cocktail, mimicking the natural process of biodegradation. This approach holds great promise for industrial applications, but targeted research is still needed on strain interactions, synergistic mechanisms, and strain pairing. Sugano et al. (2021) found that combining the white-rot fungus, *Bjerkandera adusta*, with one brown-rot fungus, *G*. *sepiarium* or *Antrodia sinuosa*, in co-cultivations displayed synergistic glucosidase and galactosidase activities. Co-cultivation of *T*. *reesei* and *Monascus purpureus* upregulated the expression of hydrolytic enzymes compared with their single cultivation. The obtained crude enzymes exhibited significant efficiency in hydrolysing wheat straw (Fatma et al. 2021). Ming et al. (2019) selected 31 strains to co-culture on distiller’s grain, among which *A*. *niger* co-cultured with *P. chrysosporium* or *T*. *reesei* presented higher activities of glucosidase (2-fold) and xylosidase (3-fold). By co-culturing low glucosidase-producing *A*. *tubingensis* with high glucosidase-producing *T*. *reesei*, Intasit et al. (2023) demonstrated the enhanced biovalorization of palm empty fruit bunches. Several studies have shown that *T*. reesei co-cultures with both *A*. *niger* and other strains showed enhanced activities of some certain enzymes, while they might also cause reduced activities of other enzymes (Sperandio and Filho 2021). Shi et al. (2021) selected five white-rot fungi strains among fifteen strains and compared their ligninolytic enzyme activities in single and co-cultivation. The results demonstrated that some co-cultures had synergistic effects on ligninolytic enzymes, some showed inhibitory effects, the others had negligible effects.

### 4.3. Process optimization

Whether it is the improvement of bioreactors or the optimisation of process parameters, it is very beneficial to optimise the existing fermentation technology before industrial production. Factors that have significant impacts on a general fermentation process include fermentation mode, strain, substrate, and process parameters (e.g. temperature, pH, agitation, dissolved oxygen, moisture). Usually, the enzyme-producing strain is selected based on the target lignocellulolytic enzyme and the composition of the agricultural waste to be utilised. Then the fermentation mode is decided according to the growth pattern of the strain. The most common goals are to minimise costs and maximise output by improving operational efficiency and shortening production periods. Parametric optimisation requires a proper design of experiment (Plackett-Burman design, Taguchi design, central composite design, and Box-Behnken design etc.) to establish validity, reliability, and replicability (Wang et al. 2020). Jana et al. (2018) produced a multi-tolerant mannanase from copra meal by *Aspergillus oryzae* and optimised the moisture and pH of the SSF through central composite design, showing a 4.3-fold increase in mannanase production (434 U/gds). Due to the diversity and complexity of agricultural feedstocks, even using the same waste, it is possible to vary the culture composition by changing the biomass size, composition and supplements. Such changes can improve raw material utilisation and productivity without greatly changing the production process and cost. Thamvithayakorn et al. (2019) performed Plackett-Burman design to screen medium components for cultivating *Pseudolagarobasidium* sp. on oil palm decanter cake. Compared to non-optimised production, the laccase, and the manganese peroxidase activities were enhanced by 2.59-fold and 1.94-fold, respectively. Gao et al. (2023) planned a Box-Behnken design with substrate size, substrate ratio, and temperature, for the SSF of *Chaetomium globosum* on agricultural wastes. The optimised conditions greatly promoted the activities of several hydrolytic enzymes, namely xylanase (by 53.67%), endoglucanase (by 95.38%), and exoglucanase (by 33.43%). Further enhancement of production can be achieved by simultaneously optimising process conditions and medium components. One-factor-at-the-time analysis was used to optimise temperature, initial moisture, and supplemental nutrients for the fermentation of pineapple crowns by *T*. *versicolor*, resulting in a 6.7-fold increase in laccase activity (Backes et al. 2022). Taguchi design was applied to optimise raw material size, supplemented galactose and cupric sulphate concentration, inoculum size, temperature, and pH for laccase production on pineapple leaves by *Marasmiellus palmivorus*. Under optimised conditions, the yield of laccase increased 17.6-fold and reached the activity of 667.4 IU/mL (Chenthamarakshan et al. 2017).

## 5. Strain modifications of fungi

The fungi-derived lignocellulolytic enzymes from agricultural wastes contain numerous kinds of degrading enzymes, corresponding to the complex structural and chemical composition of the feedstocks. These enzyme systems work synergistically in the depolymerisation of lignocellulose. However, it is relatively rare for a wild-type strain to produce exactly the desired enzyme set with the activity levels required in industry. The high enzyme cost due to low efficiency and productivity remains a major barrier in its industrial application. Strain modification approaches can be targeted to improve the enzyme production level and enzyme selectivity in fungi.

### 5.1. Homologous expression

In homologous expression, proteins are derived from their original host, have better compatibility with the host, and are more likely to obtain higher expression levels. There is significant preference in the codon coding of genes in different species, and even in genes in the same species but with different functions. Thus, homologous expression requires fewer genetic modifications. The recombinant strains are not strictly considered transgenic, making them more acceptable for use, especially in food industry. Gao et al. (2017) obtained an 9.1-fold and 51.5-fold increase in cellulase and xylanase from engineered *Penicillium oxalicum* (*P*. *oxalicum*) by overexpressing a mutated transcription factor XlnR^A871V^, a cellulase transcriptional activator ClrB and two major cellulase genes *cbh1–2* and *eg1*. Separate engineering of transcriptional activators of *P*. *oxalicum* was reported to improve different sets of lignocellulolytic enzyme productions, and combined engineering of all three activators, ClrB, XlnR, and AraR, generated 3.1-fold to 51.0-fold increases in enzyme volumetric activities of exoglucanase, arabinofuranosidase, galactosidase, xylanase, and xylosidase (Gao et al. 2021). Zhou et al. (2018) engineered *Kluyveromyces marxianus* by adding a T(−361)A mutation inside the inulinase promoter, deleting the AT-rich region inside 5’UTR (UTR∆A), and substituting P10L in the signal sequence. The recombinant strain showed up to 3-fold increased expressions of endoglucanase, xylanase, and mannanase. Liu et al. (2017) deleted up to four genes, including *cre-1*, *res-1*, *gh1–1*, and *alp-1*, from the cellulase production pathway in *M*. *thermophila* with CRISPR/Cas9 genome-editing system, resulting in over 5-fold higher cellulolytic enzyme activities. Wang et al. (2018) identified and modified a putative transcription regulator of cellulolytic enzymes, MHR1, in *M*. *thermophila*, leading to higher activities in exoglucanase (1.33-fold), endoglucanase (1.65-fold) and xylanase (1.48-fold).

### 5.2. Heterologous expression

Heterologous expression is the introduction of a gene from one species into another species. In most cases, the host strain selected for heterologous expression is generally regarded as safe and well-known for its fermentation and purification processes. These characteristics help to avoid potential risks caused by the use of pathogenic hosts, improve production efficiency, and save costs. On the other hand, the heterologously expressed proteins may lose activity or stability due to the significant differences in the codon coding between different species. Common approaches to address this issue are to optimise the codon composition without altering the amino acid sequence or expressing the gene in a heterologous host with a similar codon bias as the original host. It has been proposed that the lignocellulolytic enzyme genes from fungi can be cloned and expressed in both *Escherichia coli* and eukaryotic hosts such as *Saccharomyces cerevisiae*, *Scheffersomyces stipitis* (formerly *Pichia stipitis*) and filamentous fungi. Table 2 listed some applications of heterologous expression for lignocellulolytic enzymes and properties of the recombinant enzymes.

**Table 2.** Heterologous expressions for lignocellulolytic enzymes and properties of the recombinant enzymes.

Enzyme	Source	Gene	Host organism	Genetic modification	Properties of recombinant enzyme	Reference
Laccase	*Pycnoporus sanguineus*	*pslcc*	*Aspergillus nidulans*	Introduced heterologous gene; overexpressed transcription factor RsmA with *aflR* promoter	Promoted production (15-fold)	Li et al. 2019
	*Coprinopsis cinerea*	Lcc9	*Pichia pastoris*	Introduced heterologous gene	Higher specific activity (3.4-fold), increased stability in pH 4.5–9, improved dye decolourisation	Xu et al. 2019
Lignin peroxidase	*Thermothelomyces thermophilus*	LiP	*Aspergillus nidulans*	Introduced heterologous gene	Higher enzyme purity, large-scale fed-batch production in stirred-tank bioreactor	Liu et al. 2021
	*Phanerochaete chrysosporium*	LiPH8	*Pichia pastoris*	Introduced heterologous gene; optimised condon sequence	Higher activity, enhanced production in 14 L bioreactor, upgraded technical lignin	Majeke et al. 2020
	*Phanerochaete chrysosporium*	LiPH8	*Escherichia coli BL*21 (DE3)	Introduced heterologous gene	Easy purification, efficient and effective decolourisation of melanin	Sung et al. 2019
Endoglucanase	*Aspergillus fumigatus*	DBiNU-1	*Kluyveromyces lactis*	Introduced heterologous gene	High stability at pH 4–8 and 30–60 °C	Rungrattanakasin et al. 2018
Endoglucanase	*Fervidobacterium* sp. (77% identity)	Cel776	*Saccharomyces cerevisiae*	Introduced heterologous gene	Thermophilic, stable in presence of detergent, easy purification with high yield	Escuder-Rodríguez et al. 2022
Glucosidase	*Aspergillus niger*	*bgl*	*Pichia pastoris*	Introduced heterologous gene	High level production (129 IU/mL) in 1 L fermenter	Xia et al. 2018
	*Talaromyces amestolkiae*	*bgl-1*	*Pichia pastoris*	Introduced heterologous gene	High purification yield (>80%), high selectivity	Méndez-Líter et al. 2020
Xylanase	*Trichoderma reesei, Neurospora crassa*	Xyr1, XLR-1	*Penicillium oxalicum*	Introduced and mutated heterologous genes	Increased cellulase production (2.8-fold)	Xia et al. 2019
	*Thermothelomyces thermophila*	*ex30a*	*Pichia pastoris*	Introduced heterologous gene	Endo-action similar to bacterial xylanases, exhibited an exo-activity	Katsimpouras et al. 2019
Arabinanase	*Thermothielavioides terrestris*	Abn93T	*Aspergillus nidulans*	Introduced heterologous gene	Thermophilic, optimum activity at 70 °C, prolonged stability in pHs 4.0–6.5	Velasco et al. 2020
	*Rasamsonia emersonii*	*Reabn93*	*Pichia pastoris*	Introduced heterologous gene	Thermophilic, maximum activity at 70 °C for 3 h, stable at pH 3–9	An et al. 2022
Glucuronidase	*Thermotoga petrophila*	*Tpgus*	*Escherichia coli* BL21 CodonPlus (DE3)-RIPL	Introduced heterologous gene	Improved thermophilic activity (7-fold), great thermostability at 85 °C for 12 h	Ul and Akram 2019
Endoglucanases, glucosidase, xylanase, xylosidase, acetylxylan esterase	*Trichoderma reesei*, *Aspergillus niger*, *Aspergillus oryzae*, *Chrysosporium lucknowense*, *Talaromyces emersonii*, *Clostridium cellulovorans*	*bgl1*, *xlnD*, *xlnB*, *celB*, *cb2b,cbh*, and *xynA*	*Saccharomyces cerevisiae*	Introduced and integrated seven heterologous genes; optimised condon sequence	Enabled simultaneous saccharification and fermentation of lignocellulose into ethanol without separate enzyme hydrolysis	Claes et al. 2020

## 6. Conclusions and perspectives

**Figure 2.**
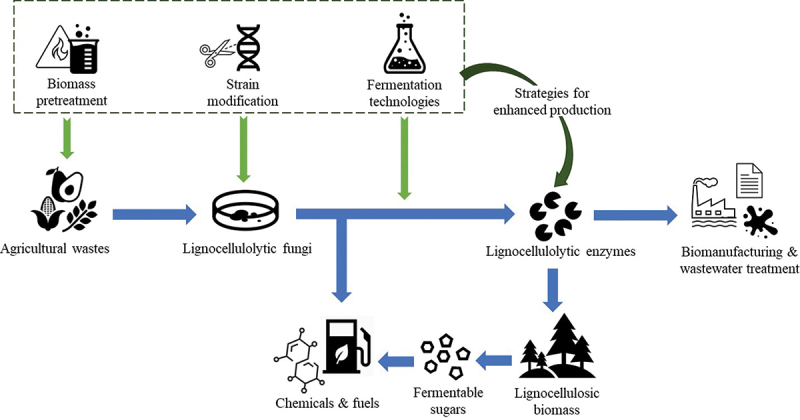
Several production strategies support the fungi-derived lignocellulolytic enzyme production from agriculture wastes and promote enzyme applications.

Agricultural waste is a sustainable and promising feedstock for microbial enzyme production. Recent researchers have achieved great progress in producing fungi-derived lignocellulolytic enzymes from various agricultural wastes as well as optimising the composition and activities of the enzyme cocktails. Since the composition of different agricultural wastes varies greatly, identifying the suitable strain for a specific feedstock is the key to obtaining efficient enzyme production. In addition, the inhomogeneity and randomness of the agricultural residues may pose more challenges to large-scale production. Although biomass pretreatment is beneficial to high enzyme yield, it is critical to explore the balance between energy-intensive pretreatment and productivity improvement. Advanced fermentation technologies and modern strain modification techniques have brought fungal lignocellulolytic enzyme production to a new stage. However, multiple aspects of fungal enzyme production still require further study, particularly fungal metabolic pathways, process control, and enzyme selectivity. Overall, identifying and applying the appropriate production strategies will significantly promote fungi-derived lignocellulolytic enzyme production from agricultural wastes. The enhanced large-scale production of lignocellulolytic enzymes presents great potential for the industrial bioconversion of lignocellulosic materials into value-added compounds (Figure 2).
